# Anti-tumor antibody isotype response can be modified with locally administered immunoadjuvants

**DOI:** 10.1007/s13346-022-01258-8

**Published:** 2022-11-23

**Authors:** Adam A. Walters, Abrar Ali, Julie Tzu-Wen Wang, Khuloud T. Al-Jamal

**Affiliations:** Institute of Pharmaceutical Science, Faculty of Life Sciences & Medicine, King’s College London, Franklin-Wilkins Building, 150 Stamford Street, London, SE1 9NH UK

**Keywords:** B16F10, CpG, Immunoadjuvants, In situ vaccination, Isotype, Antibody neutralisation

## Abstract

In situ vaccination with immunostimulatory compounds is a demonstrated means to treat tumors preclinically. While these therapeutic effects have been attributed to the actions of T cells or innate immune activation, characterisation of the humoral immune response is seldom performed. This study aims to identify whether the injection of immunoadjuvants, Addavax (Adda) and cytosine-phosphorothioate-guanine oligodeoxynucleotide (CpG), intratumorally can influence the antibody response. Specifically, whether intratumoral injection of immunoadjuvants can alter the tumor-specific antibody target, titre and isotype. Following this, the study aimed to investigate whether serum obtained from in situ vaccinated mice could neutralise circulating tumor cells. Serum was obtained from mice bearing B16F10-OVA-Luc-GFP tumors treated with immunoadjuvants. Antibody targets’ titre and isotype were assessed by indirect ELISA. The ability of serum to neutralise circulating cancer cells was evaluated in a B16F10 pseudo-metastatic model. It was observed that tumor-bearing mice mount a specific anti-tumor antibody response. Antibody titre and target were unaffected by in situ vaccination with immunoadjuvants; however, a higher amount of IgG2c was produced in mice receiving Adda plus CpG. Serum from in situ vaccinated mice was unable to neutralise circulating B16F10 cells. Thus, this study has demonstrated that anti-tumor antibody isotype may be modified using in situ vaccination; however, this alone is not sufficient to neutralise circulating cancer cells.

**Graphical Abstract:**

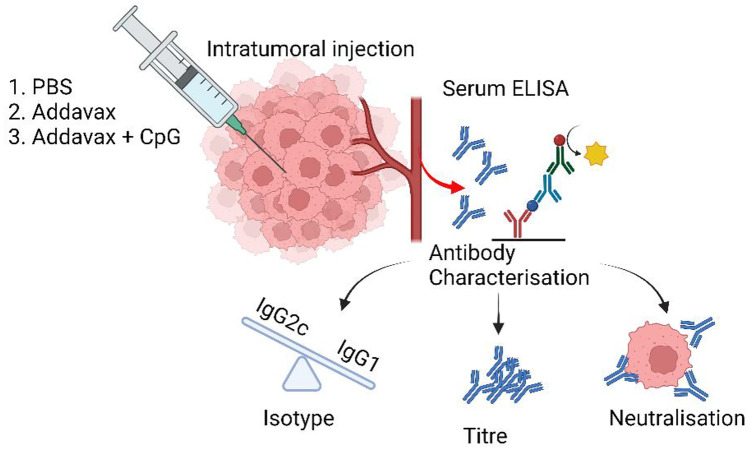

## Introduction

Immunotherapy is based on mobilising the patient’s immune system to treat cancer. There has been extensive research into immunotherapy including the development of CAR-T, checkpoint inhibitors and antibody drug-conjugates. However, limitations of these therapies are numerous and include resistance, efficacy in only a subset of patients, and cancer recurrence [1]. In situ vaccination involves injecting immunoadjuvants and immunomodulators intratumorally; these localised immune responses may initiate tumor cell death while also potentiating systemic immune responses [2]. While there have been descriptions of the role of T-cells in in situ vaccines, there has been a lack of research into whether this approach can be used to modify antibody/humoral responses [2, 3].

Humoral immunity in cancer is complex; there is conflicting evidence on the function of B-cells. In some cases, B-cells are thought to have an immunosuppressive, regulatory function [4]. For instance, it has been shown that temporarily depleting B-cells results in an increase in anti-cancer T-cell responses [5]. In contradiction, others found that B-cell depletion resulted in enhanced B16 melanoma growth, metastasis and decreased CD4 and CD8 levels [6]. Studies have also found that total depletion of B cells results in an increase in T-cell activity and a type 1 immune response; however, despite this, the presence of B-cells had a positive role in anti-cancer activity [7]. Therefore, the humoral immune response may be an essential component of an anti-tumor immune response, resulting in the production of antibodies, which can stimulate effector cells and reduce disease progression [8].

Indeed, the use of recombinant, exogenously administered, anti-tumor antibodies is proven to be a successful strategy in cancer therapy. Trastuzumab, a recombinant humanised monoclonal antibody of the IgG1 isotype, which recognises the HER2 receptor overexpressed on some breast cancers, has been shown to improve survival rates in patients with early-stage HER2-positive breast cancer [9]. Trastuzumab induces apoptosis through antibody-dependent cellular cytotoxicity in metastatic breast cancer [10]. A major limitation of exogenously administered antibody drugs is that they only work in a small number of patients and are dependent on antigen expression levels. Inducing the production of endogenous antibodies through in situ vaccination may circumvent this shortcoming, providing a source of patient-specific antibodies. In theory, these in situ generated antibodies may enhance cytotoxic T-cell responses, neutralise circulating tumor cells and prevent dissemination.

However, as with the B-cell responses, the role of endogenous antibodies in cancer is complex. There has been some debate as to whether antibodies have a pro- or anti-tumor effect. Antigen-specific antibodies form immune complexes, which are thought to activate granulocytes and macrophages, resulting in an inflammatory immune response causing enhanced tumor invasion and metastasis [11]. A study found that antibodies can promote neoplastic progression and cause carcinoma development, thought to be due to the activation of Fc receptors on leukocytes in neoplastic tissue [12]. In contrast, Carmi et al. identified allogenic tumor rejection in mice through the production of antibodies. Furthermore, when the antibodies were mixed with dendritic cells and reinjected, mice could resolve metastases and the primary tumor [13]. Clinically, the presence of anti-MUC1 IgG antibodies has correlated with improved disease prognosis in pancreatic cancer [14].

In theory, due to the distribution and function of endogenous antibodies, they may be able to prevent cancer cell dissemination, limiting invasion and secondary metastasis. In agreement, Parratto et al. found, preclinically, that poorly metastatic tumors induce a higher antibody response than non-metastatic variants of the same cell line [15]. Furthermore, clinically, it has been shown that circulating antibodies are higher in patients with non-metastatic melanoma compared to metastasised disease and higher titres are correlated with less severe disease [16]. In agreement, it has been shown that patients with non-metastatic melanoma had a higher antibody titre than those with metastatic disease, suggesting antibody levels are reduced during disease progression [8]. However, it should be noted that these are correlative observations only, and the direction of the correlation or causal link is yet to be confirmed. While antibody titre has been assessed and correlated to non-metastatic disease, the quality of these antibody responses in terms of antibody isotype has not been assessed. This may account for some of the ambiguity in attributing a role for antibodies in the resolution of cancer. In preclinical models, it has been shown that exogenously administered IgG2 antibodies are the most potent in activating effector responses and the only isotype capable of resolving tumor challenge [17].

The current study aims to investigate the following: (1) can in situ vaccination with adjuvants designed to boost antibody responses in infectious disease vaccines enhance endogenous anti-tumor antibody titre, (2) can in situ vaccination of immunoadjuvants skew antibody responses towards a more favourable IgG2 isotype and (3) whether serum from in situ vaccinated mice can neutralise circulating tumor cells in the blood. Adjuvants selected include Addavax (Adda, comparable to MF59), an immunoadjuvant known to increase the antibody titre and induce a mixed IgG1:IgG2 response [18], and Adda + CpG, where CpG is a TLR9 agonist known to skew the antibody response towards the IgG2a isotype (equivalent to IgG2c in C57BL/6 mice) [19]. The use of MF59 and CpG in combination has previously been demonstrated to simultaneously boost antibody titres and increase levels of IgG2c in response to injected tumor antigen [20]. It was hypothesised that in situ vaccination with immunoadjuvants will increase the titre of tumor-specific antibodies, while the inclusion of CpG may skew the antibody response towards IgG2c.

## Materials and methods

### Materials

Nunc Maxisorp™ flat-bottom plates (ELISA plates) and Bicinchoninic acid assay (BCA) were obtained from Thermo Fisher Scientific (UK). The secondary antibodies were horse radish peroxidase (HRP)-conjugated Goat anti-Mouse IgM, and Goat anti-Mouse IgG1 obtained from BIO-RAD, Anti-Mouse IgG HRP-linked antibody obtained from Cell Signalling Technology (UK) and Goat Anti-Mouse IgG2c-HRP antibody obtained from SouthernBiotech (USA). Bovine serum albumin (BSA) and ovalbumin (OVA) were obtained from Sigma-Aldrich (UK). Addavax was obtained from Invivogen (France). CpG 1668 (CpG) was synthesised by Eurogentec (Belgium). Trypsin EDTA, GlutaMAX™, RPMI 1640 media, phosphate buffer saline (PBS) and fetal calf serum (FCS) were obtained from Gibco, Thermo Fischer Scientific. Tetramethylbenzidine (TMB) high-sensitivity substrate solution and ELISA stop solution were obtained from BioLegend (USA). d-Luciferin potassium salt was obtained from Syd Labs (USA).

### Cells and cell culture

B16F10 were obtained from ATCC^®^, a variant of B16F10 which had been retrovirally transfected with Ovalbumin; firefly luciferase and green fluorescent protein genes was obtained from in-house sources (B16F10-OVA-Luc-GFP) [21]. Cells were cultured in RPMI supplemented with 10% FCS 1% v/v Glutamax, 50 U/ml penicillin and 10 µg/ml streptomycin. Cells were typically passaged 2–3 times weekly by first removing culture media and then washing the cell layer with PBS. Trypsin EDTA was used to dissociate cells at 37 °C at which point they were split (1:5–1:10 ratio). Cells were cultured in a humidified 37 °C, 5% CO_2_ incubator.

### Animals

Animal experiments carried out complied with current personal as well as project licences issued by the UK home office and conforming with the UKCCCR guidelines (1998). Envigo (UK) was the distributor of female C57BL/6 mice with ages ranging between 4 and 6 weeks.

### In vivo studies

B16F10-OVA-Luc-GFP (1 × 10^6^ cells per mouse in 100 µL) were implanted subcutaneously into the lower right flank of C57BL/6 mice. At days 5 and 11 post implantation, tumors were treated intratumorally with either PBS, Addavax (25 µL per mouse), CpG (25 µg per mouse) or a combination of Addavax and CpG in a total volume of 50 µL per mouse. Addavax was used at a dose based on supplier’s data sheet whereas CpG was used at a dose comparable to previously published in situ vaccination regimes [22, 23]. The growth of the tumors was monitored every 2–3 days. The experiment was terminated when tumors of the PBS-treated group reached 14 mm in diameter. Naïve serum was obtained from non-tumor-bearing mice.

### Identification of antibody targets and isotypes

The antibodies found in the serum were analysed using indirect enzyme-linked immunosorbent assay (ELISA) to identify the antibody titre, target and isotype. The cells of interest were obtained from tissue culture. Cells (1 × 10^7^) were dispersed in 10 mL of PBS; 100 μL was pipetted into each well (1 × 10^5^ cells per well) of an ELISA plate. The plate was covered and incubated at 37 °C and 5% CO_2_ for 24 h. For detection of antibodies against OVA protein, 100 μL of 5 mg/mL ovalbumin solution was added into each well. After the 24 h, each well was blocked using 200 μL of blocking buffer which consisted of 2% BSA in PBS for 1 h at room temperature. The plate was then washed using 200 μL of PBS 5 times. Following this, 100 μL of the mouse serum diluted 1:40 to 1:5000 in blocking buffer was added to the appropriate well. Where no serum was added, blocking buffer was used as a blank. The plate was then incubated at room temperature for 2 h prior to washing as described. Following this, 100 μL of the secondary antibody was added. This was either anti-mouse IgG, IgM, IgG1 or IgG2c HRP-linked antibody diluted at 1:2000 in blocking buffer. The plate was incubated at room temperature for 1 h and washed as described. To develop the plate, 50 μL of TMB was added, and incubated for between 5 and 15 min. To stop the reaction, 50 μL of stop solution was added. The optical density was obtained by reading with a FLUOstar omega microplate reader (BMG Labtech, Germany) at optical density (OD) 450 nm and 512 nm (background absorbance). Data was presented as optical density with the background and the mean of the blank values subtracted.

### Antibody neutralisation study

To establish a pseudo metastatic model B16F10-OVA-Luc-GFP (2.5 × 10^5^ per mouse) were mixed with 50% serum comprising of a pool derived from mice with either a high IgG1 (*n* = 5 mice from PBS group) or a high IgG2c (Adda *n* = 2 and Adda + CpG *n* = 3 group) in 100 µL per mouse (50 µL serum, 50 µL PBS) and was incubated for 30 min at 4 °C. As a control, an additional group was included in which cells were incubated in PBS only. C57BL/6 mice were injected intravenously into the tail vein with 100 μL of the cell-serum mix. On day 11 post injection, the mice were sacrificed, and the lungs were obtained. The number of nodules on the lungs was counted using a light microscope. To extract the luciferase, the lungs were suspended in lysis buffer and homogenised using T 18 digital ULTRA-TURRAX (IKA, UK). The homogenate was then freeze thawed twice at − 80 °C. To quantify the amount of luciferase found in the lungs, luciferin was added automatically, and the light emission was read using the FLUOstar omega microplate reader (BMG Labtech, Germany). To normalise luciferase activity to protein content, the quantity of protein extracted was assayed using a BCA protein assay. In brief: homogenised lung was diluted twofold down the plate. The BCA working reagent was added at 200 μL to each well and incubated for 10 min at 37 °C. The plate was read by FLUOstar omega microplate reader (BMG Labtech, Germany) at 562 nm. BSA was used as a standard starting at a concentration of 2 mg/mL, while PBS was used as a blank. Protein values were obtained by interpolation of blank subtracted values from a standard curve. Data is presented as relative light units divided by the protein content and is expressed as light units per milligramme of protein.

## Results

### Mice are able to mount an anti-tumor antibody response

**Fig. 1:**
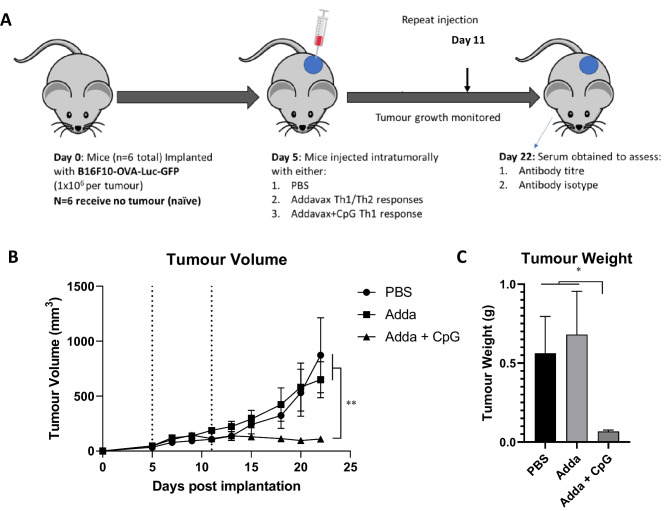
Mouse serum was obtained from mice following therapeutic intervention. C57BL/6 mice were implanted with B16F10-OVA-Luc-GFP (*N* = 6 per group did not receive a tumor (naïve)) (**A**). On days 5 and 11, 6 mice were injected with PBS, Addavax (25µL) and Addavax (25µL) with CpG (25 µg) respectively. Tumors were measured every 3–4 days and tumor volume is reported in (**B**). On day 22, mice were sacrificed, and blood was taken for sera analysis. Tumors were excised and weighed (**C**). Each point represents the mean ± SEM statistical analysis was performed using two-way ANOVA or Student’s *T* test *p < 0.05 **p < 0.01

**Fig. 2:**
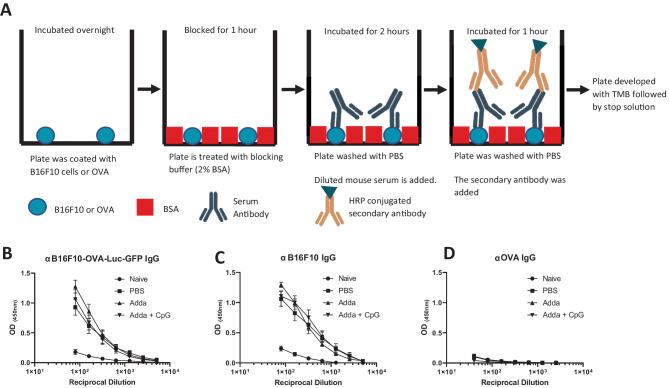
B16F10 cells are the target for antibodies following tumor challenge and antibody titre is unaffected by local administration of the immunoadjuvant. Antibody levels in serum obtained from mice bearing B16F10-OVA were detected using ELISA. ELISA plates were first coated with target antigen followed by blocking using 2% BSA. Following this, serum was added at a range of dilutions ranging from 1:40 to 1:5000. Serum was incubated for 2 h before being washed. Anti-mouse IgG HRP conjugate was added as the secondary antibody and the plate was incubated for 1 h. Following this, the TMB was added and the plate was left to develop (**A**). The stop solution was added and the absorbance was measured at 450 nm using FLUOstar omega microplate reader. The OD values obtained when either the homologous tumor cell line, B16F10-OVA-Luc-GFP (**B**); the untransformed cell type, B16F10 (**C**); or OVA protein (**D**) was used as target antigen. Points represent the mean of *n* = 6 mice per group. Error bars represent the standard error of mean

Serum was obtained from naive mice or mice bearing B16F10-OVA-Luc-GFP tumors treated intratumorally with PBS, Adda or Adda + CpG as outlined in Fig. 1A. As shown in Fig. 1, treatment of tumors with Adda had no effect on tumor growth; however, when CpG was included into the regime, tumor growth was statistically reduced as illustrated by lower end tumor volume and weight. To investigate whether the therapeutic interventions had modified the humoral immune response, serum was analysed using indirect ELISA with whole cells as the coating antigen. Gentle washing was performed between each step avoiding the use of surfactant to minimalize disruption of cell membrane/lipoproteins (Fig. 2A). As shown in Fig. 2B, when B16F10-OVA-Luc-GFP was used as a coating antigen, all groups which had tumors mounted a specific antibody response. There was some background from the naïve group; however, this may be due to the relatively high concentration of serum utilised. Though the Adda group had the highest average titre, there were no significant differences between groups strongly suggesting that antibody titre is unaffected by in situ vaccination. To establish whether the target of the antibody responses was the tumor cell line or the major foreign antigen, OVA, expressed by B16F10-OVA-Luc-GFP, ELISA plates were coated with either untransformed B16F10 or OVA protein. A near identical trend in antibody binding and titre was observed when the untransformed cell line, B16F10, was used as the coating antigen (Fig. 2C) compared to B16F10-OVA-Luc-GFP. Likewise, there were no statistical differences between treatment groups. Notably, there were no IgG antibodies specific for ovalbumin protein detected above background (Fig. 2D). The target of the antibody responses was therefore established to be the B16F10 cells rather than the OVA transgene.

### The anti-tumor antibody response is predominantly IgG1 though IgG2c levels can be increased by in situ vaccination

**Fig. 3:**
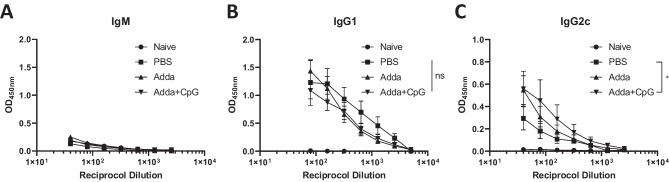
Antibody isotype is altered following therapeutic intervention. To identify the isotype of the mouse antibodies, ELISAs were carried out as previously described. However, in place on anti-mouse total IgG, either anti-mouse IgM, IgG1 or IgG2c HRP conjugates were used. The OD values obtained for each of these are shown in **A**, **B** and **C** respectively. The points represent the mean of *n* = 6 mice and the error bars correspond to the standard error of mean. Statistical analysis in graphs was carried out using a two-way ANOVA *p < 0.05, ns non-significant

Previous research has shown that different antibodies have different functions in the body. According to the hypothesis, CpG skews the antibody isotype towards IgG2c. Isotype identification of the anti-B16F10 antibodies in the mouse serum became the next interest. This was investigated using an indirect ELISA as outlined in Fig. 2A; however, specific anti-mouse IgG1, IgG2c or IgM HRP conjugated secondary mAb were utilised. No anti-B16F10 IgM antibodies were found in the serum above background levels (Fig. 3A). A high level of anti-B16F10 IgG1 was detected in all tumor-bearing groups, with OD exceeding 1 in all cases, suggesting this is the dominant anti-tumor antibody response. Again, Adda had the highest mean titre, followed by PBS and Adda + CpG, though the differences were non-significant due to high intra-group variability. Comparably, IgG2c was detected in all tumor-bearing groups however at a lower level compared to IgG1, with mean ODs not exceeding 0.6. Though it should be noted, care must be taken when comparing between ELISAs, as these were not standardised, differences may be due to secondary antibodies. As shown in Fig. 3C, we observed statistically higher IgG2c in mice receiving Adda + CpG compared to PBS, though not Adda alone, with mean ODs approximately double (OD 0.29 vs 0.55). The general trend was the reverse of that observed for the IgG1 response with Adda + CpG yielding the highest titre, followed by Adda, then PBS.

### Serum from in situ vaccinated mice cannot neutralise circulating cancer cells

**Fig. 4:**
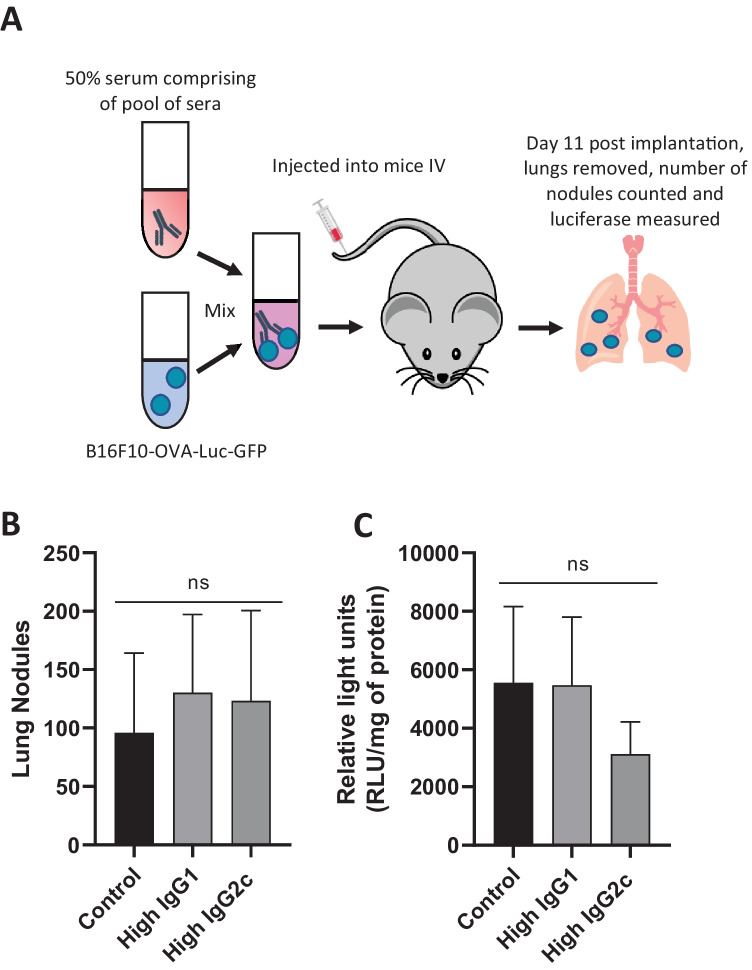
Serum of in situ vaccinated mice is unable to neutralise B16F10-OVA-Luc-GFP cells. B16F10-OVA-Luc-GFP cells were mixed with 50% serum comprising of a pool of sera derived from either mice with high IgG1, high IgG2c or (*n* = 3–4 per group). This was incubated for 30 min at 4 °C. Cells were then implanted IV into the tail vein of C57BL/6 mice. On day 11, the mice were sacrificed and the lungs were taken (**A**). The lungs were observed using a stereoscopic microscope and the number of B16F10 nodules were counted. The mean number of metastasise (*n* = 3–4) is shown in **B**, where the error bars correspond to the standard deviation. The lungs were then frozen and thawed twice at − 80 °C. Following this, the lungs were homogenised in PBS. The mean luciferase extracted was normalised to the amount of protein as calculated by BCA assay is shown in **C**, where the error bars correspond to the standard deviation. Statistical analysis was carried out using an ordinary one-way ANOVA, ns non-significant

To identify whether antibodies could neutralise circulating tumor cells, a pseudometastatic mouse lung cancer model was used. As outlined in Fig. 4A, B16F10-OVA-Luc-GFP cells were mixed with a pool of serum obtained from mice with either a high IgG1 (High IgG1) or high IgG2c (High IgG2c) titre and injected intravenously. On day 11 post implantation, the mice were sacrificed, and the lungs were removed, both lung nodules and luciferase were measured. As shown in Fig. 4B, the number of nodules found on the lungs was not significantly different between groups due to the large intra-group variation. This strongly suggests that the coincubation with serum had no effect on lung colonisation under the protocol conditions used, i.e. serum volume containing a specified amount of antibodies to cell number ratio. To further confirm this, luciferase in the lungs was assayed. Figure 4C shows luciferase per milligram of lung homogenate, though the mean level of luciferase is lower in the high IgG2c group; consistent with the previous data, this is not significantly different from the control due to high variability. Combining these data suggests serum obtained from in situ vaccinated mice cannot neutralise circulating cancer cells.

## Discussion

This study aimed to investigate whether in situ vaccination through the intratumoral injection of immunoadjuvants, Addavax and CpG could enhance the endogenous antibody immune response against the cell line, B16F10-OVA-Luc-GFP. It was important to firstly identify the target of the antibodies found within the serum. In accordance with the hypothesis, the mice bearing tumors had significantly higher tumor-specific IgG antibodies than the naïve mice. This study established that antibody responses are mounted against the tumor cell line rather than the transgene, as no ovalbumin-specific IgG antibodies were detected in the serum. This may be a result of ovalbumin being expressed intracellularly and was therefore an inaccessible target for a significant antibody response. C57BL/6 B16F0 melanoma cells have previously failed to produce antibodies against intracellular antigens, ovalbumin and PSA, suggesting B16F10-OVA-Luc-GFP may induce an immunosuppressive environment [24]. This is not universal and may be due to the properties of this cell line as other intracellular antigens such as p53 can induce an antibody response [25]. The effect of the transgenes in this model is unknown; however, in our own studies, we have observed B16F10-OVA-Luc-GFP tumors grow more slowly than B16F10, typically reaching end point by days 21–25 compared with day 14 (data not shown).

Clinical studies had found tumor-specific IgG antibodies in patients with melanoma suggesting the model selected is relevant [8, 16]. In this experiment, there were no significant differences observed in antibody titre between the mice treated with PBS, Adda or Adda + CpG. However, addavax has been shown to increase antigen-specific antibodies in conventional vaccines [26]. Although Adda + CpG vaccines can improve the T-cell response against tumor antigens, demonstrated by the growth delay curve obtained in this work for this combination treatment, the nature of the tumor may inhibit antibody responses to the tumor [20, 27]. Other studies have demonstrated that repeated systemic administration of immunomodulators over a month, resulted in a gradual increase in anti-tumor IgG antibodies with time [28]. This suggests had the mice been sacrificed at a later timepoint, there may have been more time for the antibodies to be generated, and therefore, a larger difference would be seen between groups. An increase in dose and dosing scheduling of CpG and Addavax may also have resulted in higher antibody titres.

Classification of antibody isotype post tumor challenge has been poorly studied. This is a topic of interest as antibody isotypes have been attributed different functions, and greater knowledge into how to modify the humoral immune response may provide novel therapeutic avenues. No IgM antibodies were detected, which may be a result of IgM antibody fundamental properties. IgM antibodies have active and integral roles in the early primary immune response. Diaz-Zaragoza et al. found that natural IgM antibodies are important for recognising and destroying precancerous and cancerous cells, while adaptive IgM antibodies are known to be the first antibody isotype to appear in an immunological challenge; the titre of the IgM antibodies drop as IgG antibodies are produced [29]. We speculate no IgM antibodies could be detected due to the low titres at this later time point. Both IgG1 and IgG2c antibodies were found in the serum of mice bearing tumors. Higher IgG2c antibodies were found in mice treated with Adda + CpG. As there was no CpG only group, and there were no statistical differences between Adda and Adda + CpG, we cannot conclude on whether this was entirely due to the presence of CpG or synergistic interactions between the two adjuvants. CpG is known to induce a Th1 type response marked by production of interferon gamma which, in turn promotes an IgG2 antibody response [30, 31]. Th1 cells are generally considered to be anti-tumoral, supporting cytotoxic T cell development and activities, while Th2 responses have been linked to increased angiogenesis and the development of an immunosuppressive micro-environment [32]. Despite this, in preclinical models, both subsets have been shown to be capable of inducing anti-tumor responses to varying degrees by distinct mechanisms [33, 34]. It is likely the effects of skewing the Th1/Th2 balance will be tumor and context specific. As the focus of this study was on investigating the antibody responses, we did not characterise the T cell response; however, it has been shown that and MF59 adjuvanted influenza vaccine injected intratumorally can induce antigen specific protective CD8 + T cells [35].

The interaction of CpG with B cells is complex, it has been shown that TLR9 is highly expressed on memory B cells and My88 signalling is required for class switching to IgG2c [36, 37]. The definitive mechanism of action of Addavax is still unknown. In a recent study, in situ vaccination with multiple modalities designed to promote T cell immunity was shown not to induce a class switch. Differences from this published study may be due to either the therapeutic agent or the assay used for measurement, in our study we used ELISA whereas they used flow cytometry [38]. Levels of IgG2b and IgG3 were not analysed in this study, though it would be interesting to observe if trends are consistent amongst all IgG isotypes in future work.

A mouse study was conducted to observe the neutralising capability of tumor-specific antibodies in serum. Initially, it was thought, the more anti-tumorgenic IgG2c antibodies would be able to neutralise the tumor cells. It should be noted that more subtle effects of the antibodies may be observed in in vitro assays; however, we deemed the in vivo model most representative of the clinical setting. In our study, we found no difference between groups, consistent with published data [38]. Other groups have demonstrated that adoptive transfer of serum from mice treated with an intense regime of systemic immunomodulators could protect mice from intravenous B16F10 challenge [28]. Whether such a potent antibody response can be induced by local delivery has yet to be demonstrated. Our study suggests that increases in beneficial IgG2c titre can occur following intratumoral treatment. Future work may seek to improve on our preliminary findings, for instance by inclusion of alternate or multiple TLR ligands, such as ligands for TLR3 which have been shown to potently increase IgG2 titres in vaccine models [39].

## Conclusion

Antibody responses following in situ treatment of tumor with immunoadjuvants have been described in terms of antigenic targets, titre and isotypes using indirect ELISA. Tumor cells were identified as the primary target of the antibodies, rather than transgenes. Immunoadjuvant in situ treatment of the tumors did not increase antibody titres. However, it was shown that treatment with Adda + CpG can increase relative IgG2c titres. In situ vaccination is therefore a viable approach to modify the nature of endogenous antibody responses and should be further investigated.

## Data Availability

All data generated or analysed during this study are included in this published article.
